# Adherence to treatment and harmful effects of medication shortages in the context of severe crises: scale validation and correlates

**DOI:** 10.1186/s40545-023-00667-5

**Published:** 2023-11-29

**Authors:** Sandrella Bou Malhab, Chadia Haddad, Hala Sacre, Aline Hajj, Rony M. Zeenny, Marwan Akel, Pascale Salameh

**Affiliations:** 1 INSPECT-LB (Institut National de Santé Publique, d’Épidémiologie Clinique Et de Toxicologie-Liban), Beirut, Lebanon; 2 Department of Natural Sciences, School of Arts and Science, Lebanese American University, Beirut, Lebanon; 3 School of Medicine, Lebanese American University, Beirut, Lebanon; 4 Research Department, Psychiatric Hospital of the Cross, P.O. Box 60096, Jall-Eddib, Lebanon; 5 Drug Information Center, Order of Pharmacists of Lebanon, Beirut, Lebanon; 6 Faculté de Pharmacie, Université Laval, Québec, Canada; 7 Oncology Division, CHU de Québec Université Laval Research Center, Québec, Canada; 8 Department of Pharmacy, American University of Beirut Medical Center, Beirut, Lebanon; 9 School of Pharmacy, Lebanese International University, Beirut, Lebanon; 10 Faculty of Pharmacy, Lebanese University, Hadat, Lebanon; 11 Department of Primary Care and Population Health, University of Nicosia Medical School, 2417 Nicosia, Cyprus

**Keywords:** Drug shortage, Medication, Financial crisis, Management, Validation

## Abstract

**Background:**

Medication shortage is a public health problem, affecting patients’ outcomes mainly through the difficulty in maintaining adherence, particularly in the context of a severe economic crisis. There is a need for a new scale that assesses the effect of medication shortage on adherence.

**Aim:**

To develop and validate a scale to evaluate the harmful impact of medication shortage among the general Lebanese population and assess its correlates and association with medication adherence.

**Methods:**

A questionnaire was used to assess medication shortage harmful effects and patients’ adherence, allowing to generate the Harmful Impact of Medication Shortage scale (HIMS). The factor analysis, convergent validity and reliability of the generated scale were assessed, followed by multivariable regressions to evaluate its correlates.

**Results:**

The developed HIMS scale is a 9-item tool, used to assess how difficult it was for people to deal with medication shortages and their harmful effects on treatment. It was significantly and inversely linked to treatment adherence and affected by the patients’ socioeconomic status and the type of chronic disease.

**Conclusion:**

The Harmful Impact of Medication Shortage scale could be an efficient tool to measure the detrimental effects of medication shortages among the Lebanese adult population with chronic diseases, particularly affecting treatment adherence. Future studies and evidence are still needed to confirm our findings and help build global mitigation policies addressing medication shortages.

## Background

The World Health Organization (WHO) defines medication adherence to treatment as “the degree to which the person’s behavior corresponds with the agreed recommendations from a healthcare provider” [1]. Adherence means that patients and physicians work together to enhance patient health by taking into account the medical opinion and patient lifestyle, values, and treatment preferences [2].

Medication non-adherence is a complicated and multifaceted healthcare issue. Non-adherent patients may decide to stop taking their medication or not to start treatment at all. Patients may also take higher or lower doses than prescribed or not respect the timing advised [3]. Thus, non-adherence can be caused by the inability of patients to take their recommended treatment due to capacity and resource constraints (problems of accessing prescriptions, medication shortages, cost, and competing demands) [4].

Furthermore, medication availability and affordability are a critical priority issue in low- and middle-income countries, where medication shortage represents a significant public health problem and affordability is considered a serious concern to any healthcare system since it affects patient health and imposes a financial burden on patients, clinicians, and healthcare systems [5]. Strategies that address medication shortage depend on the country’s economic situation and include increased reporting systems, policy changes, medication shortage platforms, and expedited medication approval [6].

Several studies and working groups have examined medication shortage mitigation and management solutions [7–9]. A recent survey, based on semi-structured interviews during the COVID-19 period in Michigan hospitals, provided insights to help develop and manage medication shortages [10]. Several studies were also conducted in Arab countries. Two explored the extent of medication shortages in Jordanian hospitals [11] and the opinion of Egyptian physicians on medication shortages [12]. The third evaluated medication shortages in large hospitals in Riyadh using the European Association of Hospital Pharmacists medication shortage questionnaire [13]. However, all these studies did not take into account patient adherence to treatment, despite the shortage issue.

Since the events of October 2019, Lebanon has been experiencing one of the world's worst economic crises, with its currency rapidly devaluating, losing more than 90% of its value. The country's health sector has been negatively impacted by the financial crisis [14], especially given that around half of the population has no health coverage, while the other half is covered by institutions also enduring financial barriers. In 2020, the COVID-19 pandemic and the Beirut port massive explosion aggravated the issue tenfold, putting even more strain on an already struggling healthcare system [14].

While unemployment and financial troubles decrease patient capacity to purchase medications, the shortage of foreign currencies has led the government to gradually lift subsidization on many essential goods, including medications, while other basic goods like food and clothing are becoming more and more expensive, and difficult to acquire [15]. The worse the crisis, the higher the shortage. Indeed, whenever the item is available, it is out of reach of the average individual. In this challenging context, many Lebanese started panic buying and hoarding available medications [14]. The situation has worsened with medications smuggled out of the country and the market flooded with substandard drugs. Consequently, many essential medications have run out, and there could be further shortages of others, while the local industry is having difficulty trying to cope with the new context. Thus, many Lebanese spend time searching the country and beyond for necessary medications [14].

Understanding medication shortages could help create a management plan that includes clear rules and processes for information collection, decision-making, cooperation, and timely communication. However, measuring adherence is critical for researchers and physicians since an inaccurate estimation can lead to several issues that are both costly and harmful. Furthermore, this measure is complex since the parameters of acceptable adherence must be carefully defined and sometimes individually tailored [16]. Several instruments are available for these measures, but they should be valid, reliable, and change-sensitive [17]. In Lebanon, the Lebanese Medication Adherence Scale (LMAS) has been previously developed to measure medication adherence, considering socioeconomic and cultural factors [18]. It was initially validated among hypertensive patients [18] and then in patients with other chronic illnesses in Lebanon [19–21], all before the current crisis and medication shortage.

Based on the above, there is a need for a new scale that assesses the effect of medication shortage on adherence. The objective of the current study is to develop and validate a scale to evaluate the harmful impact of medication shortage among the general Lebanese population and assess its correlates and association with medication adherence.

## Methods

### Study design and sampling

This cross-sectional study was conducted between January and March 2022 amidst the ongoing Lebanese economic crisis. It used an online questionnaire and the snowball sampling technique to recruit an overall sample of 350 Lebanese adults, with or without chronic diseases, using the following link: Burnout and Stigma in the Current Economic and Health Crises (google.com). The sample was then weighted for geographical dwelling region, gender, and education level, based on the Central Administration of Statistics figures to optimize sample representativeness. The current analysis is part of the larger project; it was conducted on the subsample of patients with chronic diseases (*n* = 174 participants).

### Sample size calculation

The Statcalc population survey tool of the EpiInfo™ software (Version 7, Center for Disease Control, Atlanta, USA) was used to calculate the minimum sample size for this analysis, considering an expected prevalence of medication adherence of 42% among people with chronic diseases [22], an alpha of 5%, and a beta of 20%. The minimum necessary sample was *n* = 146. The target sample size was multiplied by 1.1 to allow for possible missing values. A total of 174 participants were included in the study.

### Questionnaire and variables

The questionnaire was standardized in Arabic (the native language in Lebanon) and required 15 min to complete. The introductory section included explanations and the study objectives; participants who answered the questionnaire gave informed consent to participate implicitly. The questions covered sociodemographic characteristics, health status, and medication use aspects (e.g., adherence, barriers to obtaining), in addition to questions used to construct the scales.

#### Independent variables

The independent variables were divided into sociodemographic characteristics (such as age, gender, household income, marital status, employment status, and education level) and personal medical history (chronic diseases and medication history).

#### Major dependent variables

##### The Lebanese Medication Adherence Scale (LMAS)

The Lebanese Medication Adherence Rating Scale (LMAS) is a self-reported questionnaire validated in the general Lebanese population and used to measure medication adherence. The total score is calculated by summing all the answers; higher scores indicate lower medication adherence [18].

##### The Harmful Impact of Medication Shortage (HIMS) scale

The Harmful Impact of Medication Shortage (HIMS) scale, comprising nine items selected from previous studies [7–13, 23, 24], was developed in this study. It aims to measure the extent to which people are coping with medication shortages caused by the economic crisis in Lebanon. The tool’s questions reflect conditions experienced by the Lebanese people preventing them from being able to acquire their medications properly. These conditions include but are not limited to fuel shortage, expiry dates, and closure of pharmacies. All items are rated on a 4-point Likert scale from 1 (never) to 4 (often). The total HIMS score was calculated by summing all the answers; higher scores indicated a more harmful impact of the medication shortage. The constructed scale was assessed for adequate validity and reliability.

### Statistical analysis

Data were imported from Google Forms to an Excel spreadsheet, then analyzed using SPSS version 25.0. A descriptive analysis was first conducted to evaluate sample characteristics. The percentage of missing data was less than 5.0% of the database; therefore, no values were replaced. Construct validity of the newly developed HIMS scale was assessed in the current population using the principal component analysis technique. The Kaiser–Meyer–Olkin measure of sampling adequacy and Bartlett’s test of sphericity were calculated to ensure the adequacy of the structure. Factors with eigenvalues values higher than one were retained after a Promax rotation, and the scree plot method was used to determine the number of components to extract. Only items with a factor loading higher than 0.4 were considered. The internal consistency of the HIMS scale was assessed using Cronbach's alpha.

In the bivariate analysis, Student’s t-tests and ANOVA were used to compare means. Pearson’s correlation was applied to examine the association between continuous variables. In the multivariate analyses, many linear regressions were conducted, taking the HIMS and LMAS as the dependent variables and all the variables that showed a *p* < 0.2 in the bivariate analysis as independent variables. Linear regressions were performed according to the following model series: in the models taking HIMS as the dependent variable, the analysis included the economic conditions and effort to find the medications needed in the first model, and the health status and adherence scale were added to complete the condition. In the models taking LMAS as the dependent variable, the analysis included, in a stepwise method, the economic conditions in the first model, then the medication shortage conditions in the second model. Then these were joined in the third model. Model 4 included health status, patient characteristics influencing health status, and the HIMS scale to explain factors affecting medication adherence from different perspectives. In all cases, a p-value less than 0.05 was considered significant.

## Results

### Sample characteristics

The sample consisted of 174 participants (58.6% of females) with at least one chronic disease. Also, 46.6% were married, 25.6% had a low income, and 25% specified their chronic illnesses. The mean age of participants was 36.3 years, and the mean household crowding index was 1.4. The mean number of daily medications was around 2, with mean LMAS = 9.2 and mean HIMS = 7.2 (Table 1).

**Table 1:** Sociodemographic characteristics of the studied population (*n* = 174)

Variable	*N* (%)
Gender	
Male	72 (41.4)
Female	102 (58.6)
Marital status	
Non-married (single, widow, divorced)	93 (53.4)
Married	81 (46.6)
Monthly income	
Low income (0 to 1,500,000LBP)	62 (35.6)
High income (> 1,500,000LBP)	112 (64.4)
Type of chronic disease	
Not specified	131 (75.3)
Heart disease	12 (6.9)
Hypertension	6 (3.4)
Diabetes	7 (4.1)
Lung disease	6 (3.4)
Autoimmune disease	4 (2.3)
Other diseases	8 (4.6)

	Mean (SD**)**
Age (years)	36.28 (13.5)
Household crowding index (persons/room)	1.37 (0.82)
Number of medications taken daily (if chronic disease)	1.96 (1.45)
Number of visited pharmacies to find the needed medications	4.76 (4.24)
Lebanese Medication Adherence Scale (LMAS)	9.16 (5.78)
The Harmful Impact of Medication Shortage (HIMS) scale	7.16 (6.16)

### Factor analysis of the Harmful Impact of Medication Shortage (HIMS) scale

The HIMS best structure had eight items, loading on 1 factor with an Eigenvalue higher than 1. Items with a low loading on factors (< 0.4) or a low communality (< 0.4) were removed. The Kaiser–Meyer–Olkin measure of sampling adequacy was 0.851, and Bartlett’s test of sphericity was significant (*p* < 0.001). The extracted factor explained 61.152% of the total variance (Table 2). The reliability of Cronbach’s alpha was 0.903.

**Table 2:** Component matrix of the Harmful Impact of Medication Shortage scale

Factor	Item	Factor 1	H2 communalities
Do you stop or decrease taking your medication because the drug price has increased and you cannot afford it?	1	0.899	0.750
Do you stop buying your medication as you have other priority needs to buy?	8	0.858	0.736
Do you stop taking your medication due to the unavailability of the drug in one pharmacy?	2	0.818	0.670
Do you stop taking your medication because the nearest pharmacy is always closed?	3	0.814	0.662
Do you stop buying your medication due to a fuel shortage and you cannot reach the pharmacy?	5	0.812	0.660
Do you stop taking your medication because you cannot find the initial drug trade name that you used to take or you want?	6	0.797	0.635
Are you willing to switch to a lower dose if you cannot find the requested dose?	9	0.647	0.419
Do you stop buying your medication if the expiry date is too short?	7	0.600	0.359
Percentage variance explained		61.152%	

Cronbach alpha = 0.903

Kaiser–Meyer–Olkin (KMO) = 0.851

Bartlett’s test of sphericity *p* < 0.001

### Bivariate analysis

In our sample, the HIMS score was significantly higher among non-married participants and those with diabetes, autoimmune, and lung diseases. Higher household crowding index and higher LMAS (lower adherence) were positively correlated with the HIMS, contrary to age and the number of medications (Table 3).

**Table 3:** Correlates of the Harmful Impact of Medication Shortage (HIMS) scale

	Mean (SD)	*p*-value
Gender		
Male	10.53 (6.92)	0.094
Female	7.81 (5.09)	
Marital status		
Non-married	12.10 (6.62)	0.002
Married	6.88 (4.76)	
Monthly income		
Low income	10.00 (4.70)	0.317
High income	8.35 (7.44)	
Type of chronic disease		
Heart disease	4.11 (4.84)	0.005
Hypertension	8.39 (4.40)	
Diabetes	13.31 (5.55)	
Lung disease	10.54 (5.48)	
Autoimmune	10.39 (7.00)	
Other diseases	9.09 (4.14)	

	r	*P*-value
Age (years)	− 0.315	0.016
Household crowding index	0.571	< 0.001
Number of medications taken daily (if chronic disease)	− 0.471	< 0.001
Number of visited pharmacies to find the needed medications	0.184	0.167
LMAS (lower adherence)	0.770	< 0.001

Higher LMAS scores (lower adherence) were found in non-married individuals and those with a lower income, lung illnesses, diabetes, and autoimmune diseases. Higher LMAS (lower adherence) was also positively correlated with a higher household crowding index and a higher HIMS score (Table 4).

**Table 4:** Correlates of the Lebanese Medication Adherence Scale (LMAS)

	Mean (SD)	*p*-value
Gender		
Male	9.84 (5.53)	0.141
Female	7.70 (5.36)	
Marital status		
Non-married	11.73 (5.23)	< 0.001
Married	6.44 (4.56)	
Monthly income		
Low income	10.06 (4.72)	0.074
High income	7.46 (6.02)	
Type of chronic disease		
Heart disease	5.81 (6.12)	0.032
Hypertension	8.30 (3.26)	
Diabetes	10.60 (4.06)	
Lung disease	14.00 (7.08)	
Autoimmune	9.15 (3.65)	
Other diseases	4.51 (4.28)	

	*r*	*P*-value
Age (years)	− 0.390	0.003
Household crowding index	0.465	< 0.001
Number of medications taken daily (if chronic disease)	− 0.355	0.006
Harmful Impact of Medication Shortage scale	0.770	< 0.001

### Correlates of the Harmful Impact of Medication Shortage scale: multivariable analysis

The first linear regression, taking into account the socioeconomic status and efforts to find the medication needed, showed that a higher number of visited pharmacies to find the needed medications (Beta = 0.392) and a higher household crowding index (Beta = 4.383) were associated with a higher HIMS score, whereas a higher daily number of medication (beta = − 1.174) was associated with the lower HIMS score (borderline result; *p*-value = 0.058) (Table 5).

**Table 5:** Multivariable analysis of the Harmful Impact of Medication Shortage scale

Model 1: Linear regression taking into account socioeconomic status and efforts to find the needed medication as independent variables				
Factor	Unstandardized beta	Standardized beta	95% CI	*P*-value
Number of medications taken daily taken daily (if chronic disease)	− 1.174	− 0.274	− 2.389; 0.40	0.058
Number of visited pharmacies to find the needed medications	0.392	0.268	0.092; 0.692	0.011
Household crowding index	4.383	0.4.63	2.192; 6.574	< 0.001

Model 2: Linear regression taking into account chronic disease status and adherence to treatment as independent variables				
Factor	Unstandardized beta	Standardized beta	95% CI	*P*-value
Heart disease vs. other disease	− 6.059	− 0.435	− 9.747; − 2.373	0.002
Chronic lung disease vs. other disease	− 5.386	− 0.278	− 10.016; − 0.756	0.024
LMAS scale	0.755	0.671	0.553; 0.957	< 0.001

Variables entered: number of medications taken daily; number of visited pharmacies to find the needed medications; Lebanese Medication Adherence Scale; gender; marital status; household crowding index; age (years)

The second linear regression, taking into account chronic disease status and medication adherence, showed that a higher LMAS (lower adherence) (Beta = 0.755) was associated with a higher HIMS score, whereas heart (Beta = − 6.059) and lung (Beta = − 5.386) disease were associated with a lower HIMS score (Table 5).

### Correlates of the Lebanese Medication Adherence Scale: multivariable analysis

The first linear regression, taking into account the socioeconomic level, showed that a higher household crowding index (Beta = 2.561) was associated with a higher LMAS score (lower adherence), whereas being married (Beta = − 3.978) was associated with a lower LMAS score (higher adherence) (Table 6).

**Table 6:** Multivariable analysis of the Lebanese Medication Adherence Scale (LMAS)

Model 1: Linear regression taking into account the socioeconomic level as independent variables				
Factor	Unstandardized beta	Standardized beta	95% CI	*P*-value
Married vs. non-married*	− 3.978	− 0.362	− 7.322; − 0.635	0.021
Household crowding index	2.561	0.304	0.359; 4.763	0.023

Model 2: Linear regression taking into account the current medication shortage as independent variables				
Factor	Unstandardized beta	Standardized beta	95% CI	*P*-value
Age (years)	− 0.083	− 0.246	− 0.157; − 0.010	0.027
Harmful Impact of Medication Shortage scale	0.498	0.716	0.362; 0.634	< 0.001

Model 3: Linear regression taking into account current medication shortage and economic status as independent variables				
Factor	Unstandardized beta	Standardized beta	95% CI	*P*-value
Harmful Impact of Medication Shortage scale	0.425	0.610	0.277; 0.573	< 0.001

Model 4: Linear regression taking into account chronic disease status as independent variables				
Factor	Unstandardized beta	Standardized beta	95% CI	*P*-value
Heart disease vs. other disease*	− 5.208	− 0.421	− 8.906; − 1.510	0.007
Hypertension vs. other disease*	− 4.052	− 0.257	− 7.844; − 0.259	0.037
Chronic lung disease vs. other disease*	− 7.319	− 0.425	− 11.288; − 3.350	0.001
Age (years)	− 0.073	− 0.215	− 0.135; − 0.010	0.024
Harmful Impact of Medication Shortage scale	0.659	0.830	0.503; 0.816	< 0.001

Variables in the equation: marital status; gender; age (years); household crowding index

Variables entered: gender; age (years); Harmful Impact of Medication Shortage scale; number of medications taken daily

Variables entered: age (years); Harmful Impact of Medication Shortage; marital status; household crowding index

Variables entered: gender; age; Harmful Impact of Medication Shortage scale; type of chronic disease

*reference value

The second linear regression, taking into account current medication shortages, showed that a higher HIMS (Beta = 0.498) was associated with a higher LMAS score (lower adherence), whereas older age (Beta = − 0.083) was associated with a lower LMAS score (higher adherence) (Table 6).

The third linear regression, taking into account the socioeconomic level and current medication shortages, showed that a higher HIMS score (Beta = 0.425) was associated with a higher LMAS score (lower adherence) (Table 6).

The fourth linear regression, taking into account chronic disease status, showed that a higher HIMS score (Beta = 0.659) was associated with a higher LMAS score (lower adherence), whereas older age (Beta = − 0.073), heart disease (Beta = − 5.208), hypertension (Beta =− 4.052), and lung disease (Beta = − 7.319) were associated with a lower LMAS score (higher adherence) (Table 6).

## Discussion

In this study, the Harmful Impact of Medication Shortage (HIMS) scale could be developed and validated among a sample of Lebanese patients with at least one chronic disease. This 9-item tool is used to assess how difficult it was for people to deal with medication shortages and their harmful effects on treatment. It also evaluated the factors related to medication shortage effect and adherence to treatment.

The HIMS scale showed to be of valid structure, with high internal consistency indicating that the scale is reliable. The factor analysis of the scale revealed that all items had high loadings on one component, indicating a good factorial validity. In addition, the convergent validity with the LMAS scale was satisfactory, as the correlation analysis displayed favorable results, showing that those who responded poorly to shortages had lower adherence. These findings suggest that the scale is a valid and reliable measure for assessing the harmful impact of medication shortages in the general adult population, which might help in clinical research and practice. However, our findings could not be compared to those in the literature since no scale exists to evaluate medication shortage-related effects, and the HIMS scale was developed specifically for this study; hence, more research is necessary to confirm the validity of this newly developed scale.

The multivariable analysis showed that having heart and lung diseases was associated with a lower HIMS score and higher adherence to treatment, contrary to other illnesses; these patients may be making more efforts to cope with medication shortages, while those with other diseases are making fewer efforts. In the same direction, patients with a higher number of medications had a lower HIMS score, indicating that participants taking several medications for chronic illnesses were making more efforts to avoid worsening their health status [4]. This behavior is expected to cause fewer medication-related problems, known to occur mainly because of non-adherence to treatment [25].

An important finding of this study was that a higher harmful impact of medication shortage was associated with lower adherence to treatment. Similarly, a study among 22,830 patients surveyed at community pharmacies found that medication shortages were related to negative consequences on patients and the health system. Patients confronting barriers to purchasing medication, such as problems accessing prescriptions, cost, or unavailability of medications, might become non-adherent to treatment [6]. Patients might thus decide intentionally to stop taking a medication due to incapacity and resource limitations [26]. In Lebanon, the context fosters this problem due to economic and political instability worsened by the COVID-19 pandemic and the many challenges faced by the healthcare system, including a critical shortage of essential medications [14].

Higher numbers of visited pharmacies to find the needed medications were related to a higher harmful impact of medication shortage. Thus, many patients are tired of searching across pharmacies in Lebanon and look for other solutions to obtain their medications, despite community pharmacies being the only legal source of prescription and non-prescription medications for the population and are generally the first and last contact of patients with the healthcare system [14, 27]. Empty shelves in Lebanon community pharmacies are thus jeopardizing the role of pharmacists within the healthcare system.

Regarding the sociodemographic characteristics, our results showed that a higher household crowding index was related to less adherence and a more harmful impact of medication shortages. Overcrowded houses, well known to be in lower socioeconomic levels, are linked to various unfavorable health outcomes [28] and could be related to unintentional non-adherence to treatment. Oppositely, older age and being married were related to higher adherence to treatment. These results are similar to previous findings, showing that sociodemographic characteristics such as age, gender, marital status, and education level may affect adherence [29–31]. A systematic review that included 51 articles covering 19 different disease categories found that living with someone or being married improved treatment adherence [32]. Another study among 636 hypertensive patients revealed that being married enhanced the probability of being adherent to treatment [33]. Increased adherence might be linked to spousal help by giving practical support or improving patients' self-concept [34].

## Limitations

This study has several limitations. Its cross-sectional design limits the evaluation of cause–effect relationships. The self-reported questionnaire used to assess the harmful impact of medication shortage and adherence might be prone to information bias, underestimating the actual level of non-compliance and the impact of medication shortages. The data were gathered via a nonrandom snowball method, which may have resulted in selection bias. The mean age of the participants was low, putting them in conditions other than those of older age and thus making them prone to different types of chronic diseases seen in older patients. The sample size might not be sufficient to detect all associations and generalize to the entire population. Residual confounding bias is also possible since there might be some related factors that were not assessed in this study.

Thus, further studies are necessary to confirm the validity of the newly generated scale and the accuracy of results.

## Conclusion

Our main findings showed that the Harmful Impact of Medication Shortage scale could be an efficient tool to measure the detrimental effects of medication shortages among the Lebanese adult population with chronic diseases. In addition, the harmful impact of medication shortages was significantly related to treatment lower adherence. Future studies and evidence are still needed to confirm our findings and help build global mitigation policies addressing medication shortages.

## Data Availability

The datasets generated during and/or analyzed during the current study are available from the corresponding author on reasonable request.
